# Aging Attitudes Among Middle-Aged and Older Adults with Disabilities: Gender Differences and Predictors

**DOI:** 10.3390/geriatrics10030077

**Published:** 2025-06-05

**Authors:** Muna Bhattarai, Gloria K. Lee, Hung Jen Kuo

**Affiliations:** 1 Harris College of Nursing & Health Sciences, Texas Christian University, 2800 W. Bowie Street, Fort Worth, TX 76129, USA; 2 Department of Counseling, Educational Psychology & Special Education, Michigan State University, East Lansing, MI 48824, USA; leekalai@msu.edu (G.K.L.); kuohungj@msu.edu (H.J.K.)

**Keywords:** acceptance, aging, attitude, disabilities, mindfulness, purpose in life

## Abstract

**Background/Objectives:** Research suggests that attitudes toward aging significantly impact health and well-being outcomes in older adults and are influenced by various factors. Our study aims to identify gender differences in attitudes toward aging among aging individuals with disabilities while also examining the influence of demographic and psychological factors on these attitudes. **Methods:** For this cross-sectional study, we collected data from 393 middle-aged and older adults with disabilities via an online Qualtrics survey administered through the Prolific platform in the United States. Participants completed the Attitudes Towards Aging Questionnaire Short Form, Purpose in Life Test Short Form, Mindfulness Attention Awareness Scale, Acceptance of Chronic Health Conditions Scale, and Three-Item Loneliness Scale. Descriptive and correlation analyses, *t*-tests, and multiple regression analyses were performed. **Results:** The independent *t*-test findings reveal significant differences in physical change and psychological growth between men and women, with men scoring higher in physical change and women in psychological growth. In multiple regression analyses, purpose in life significantly predicted all three domains of attitudes toward aging in men, while both purpose in life and acceptance were predictors across all domains in women. Additionally, age, employment, and financial stability contributed to aging attitudes only among women. **Conclusions:** Attitudes toward aging, specifically physical change and psychological growth, were found to vary by gender, with purpose in life, acceptance, and loneliness influencing these attitudes among both groups, while certain demographic factors influenced aging attitudes only among women. These findings underscore the need for gender-specific interventions addressing these substantial factors.

## 1. Introduction

Aging is an inevitable process that brings numerous physical and psychosocial changes in individuals’ lives. A substantial body of research indicates that individuals’ subjective evaluation or attitudes toward aging can significantly influence their self-perception, coping strategies, and engagement in self-care practices, which in turn can affect various health and well-being outcomes among older adults [1,2,3,4]. Studies have shown that positive attitudes toward aging are associated with higher levels of life satisfaction, better mental health and cognitive functioning, lower levels of anxiety and depression [1,4], greater longevity, lower obesity rates, and better performance of activities of daily living [5]. Conversely, negative attitudes toward aging are frequently associated with poor health outcomes [2,4,5], including increased stress reactivity [3], diminished activities of daily living and recovery from disability [6], and lower quality of life [7].

Existing research and theories of intersectionality posit that a set of interrelated social factors or identities (e.g., age, gender, race, disability) determine one’s perception of their environment [8] as well as health outcomes [9,10]. Individuals’ perception of aging, which is closely associated with health outcomes, can be significantly influenced by factors such as demographic characteristics, health status, and preexisting physical limitations [1,2,11]. For instance, older adults who have higher levels of education, are employed, and self-rate their health as good tend to exhibit positive attitudes toward aging [2,11,12]. In contrast, factors such as unemployment, poor health, the presence of a greater number of chronic conditions, and increased disability are related to negative attitudes toward aging [2,13].

Importantly, intersections between individuals’ experiences of aging and disability are closely connected to how they perceive and interpret the aging process [4,14]. While aging often leads to a decline in physical abilities, the presence of a chronic illness or disability can exacerbate this process, making it more noticeable and challenging [15]. Individuals with chronic health conditions, including disabilities, often experience unique challenges as they age; thus, they are more likely to perceive aging negatively compared to those without such conditions [11]. Interestingly, older adults who attribute their illnesses to the aging process tend to experience more adverse effects from their illnesses than those who attribute their illnesses to other factors [16]. On average, individuals with fewer chronic health issues, limited functional impairments, and lower levels of chronic health-related symptoms tend to have a more positive outlook on aging [17]. One study shows that older adults with positive perceptions of aging have a 44% higher chance of achieving full recovery from severe disabilities than those who hold negative age-related stereotypes [6]. Overall, individuals with chronic illnesses and disabilities may have more negative attitudes toward aging than the general population [18].

### 1.1. Gender Differences in Attitudes Toward Aging

From an intersectional perspective, multiple social identities shape individuals’ perceptions of their health [9,10]. Consequently, attitudes toward aging are significantly influenced by a complex interplay of various factors, with notable gender differences emerging as a key identity. Previous studies indicate that women generally report more negative attitudes, including anxiety about aging, than men [12,19,20]. This difference in attitude can be attributed to several sociocultural factors, including roles and expectations that society assigns to different genders [21]. According to social role theory, attitudes and behaviors of men and women are shaped by the societal roles they fulfill, which are often accompanied by distinct responsibilities within family and society, reinforcing gender-specific attitudes that align with prevailing social norms [21,22].

While the intersectionality approach has been well integrated into health research, limited studies have examined how the intersection of gender, disability, and aging influences attitudes toward aging. Specifically, regarding the intersection of gender and aging in individuals with chronic conditions, the literature suggests that the higher incidence of chronic illnesses and disabilities among older women often results in prolonged institutionalization and a greater reliance on caregivers, whereas men typically receive care from a spouse. In addition, the relationship between gender and aging is further complicated by the notion of body image, with age-related changes in physical appearance tending to affect women more profoundly than men [19]. In a previous study, men reported more positive attitudes in the physical change domain, indicating that aging-related physical changes did not hinder their activities. In contrast, women showed more positive attitudes in the psychological growth domain, particularly regarding gaining wisdom and experiencing differences in life [19].

### 1.2. Psychological Factors Influencing Attitudes Toward Aging

Besides demographic characteristics and health-related factors, individuals’ psychological factors and resources might influence how older adults with chronic conditions perceive aging. Rowe and Kahn [23] emphasize three essential characteristics that contribute to successful aging: low risks of disease and disability, high levels of mental and physical functioning, and active engagement in life. Acceptance, mindfulness, and purpose in life are closely related to the concept of active engagement. Additionally, Flood’s [24] theory of aging further elucidates the role of intrapsychic factors, self-acceptance, and gerotranscendence (i.e., meaning and purpose in life) in shaping the perception of successful aging. Since attitudes toward aging are strongly linked to successful or unsuccessful aging [24], these factors or components of successful aging also tend to shape attitudes toward aging.

Acceptance of chronic conditions is a crucial aspect of an individual’s coping process, facilitating adjustment to new realities and fostering new positive attitudes [25]. Poor acceptance of chronic conditions has been associated with physical fatigue, social challenges, and loneliness among older adults with chronic obstructive pulmonary disease, potentially leading to negative attitudes [26]. Conversely, older adults with late-onset disabilities, who adapt to age-related changes by accepting their disabilities as an inherent part of the aging process, often demonstrate a positive perception of aging [14] and better health [27,28]. Research also suggests the role of purpose in life in shaping one’s aging attitude. A strong sense of purpose in life was reported to mitigate the impacts of health-related risk factors, promoting positive aging attitudes and thus improving subjective well-being [29,30,31,32]. Additionally, mindfulness appears to help individuals in accepting their aging process and associated disabilities with a positive mindset [33,34,35]. Wang et al. [36] noted positive longitudinal relationships between mindfulness and attitudes toward aging in promoting successful aging among older adults. Furthermore, mindfulness intervention has shown promising results in improving attitudes toward aging [37]. While acceptance, purpose, and mindfulness are likely to promote positive attitudes, loneliness seems to contribute to negative attitudes toward aging. As loneliness is more prevalent among older adults, its association with anxiety and depression may significantly determine their perceptions of aging [38,39]. Older adults experiencing loneliness are more likely to report more negative attitudes than those who live with others [12].

While various studies have examined the attitudes toward aging in the general population, there remains a notable gap in research focused on how gender and disability intersect with aging to impact these attitudes. Understanding the influence of gender is important, as the findings could provide valuable insights, enabling researchers and practitioners to design more effective interventions tailored for diverse groups. Moreover, the role of psychological factors in shaping these attitudes among individuals with disabilities deserves more attention. Therefore, the present study aims to (1) examine gender differences in attitudes toward aging among middle-aged and older individuals with disabilities and (2) assess the influence of demographic and psychological factors such as purpose in life, mindfulness, acceptance, and loneliness on these attitudes.

## 2. Materials and Methods 

### 2.1. Design and Participants

For this cross-sectional study, we used G*Power version 3.1 to estimate sample size based on the data analysis plan. Consequently, a sample size of 160 is recommended for multiple regression analysis given a medium effect size (f^2^ = 0.15) and the relevant predictors. We decided to oversample to account for potential missing data and address other research questions. A total of 400 individuals living with disabilities across the United States participated in the online survey. Upon review of the complete dataset, no missing data was observed. Among the participants, 143 (35.8%) were identified as men, 250 (63.6%) as women, and seven (1.8%) as others. Due to the small sample size for other gender categories, our analysis in this paper included exclusively 393 participants identifying as men or women.

The mean age of the 393 participants was 60.36 (SD = 7.73), with a range of 50 to 95 years. The majority of participants were below 65 years of age (72.8%) and White (86.5%), followed by Black (6.4%), with Puerto Rican participants representing the smallest percentage (0.3%). Of the participants, 46.6% were married or living with a partner, while 4.9% of men were widowed. Among those who attended college but did not obtain a degree (25.2%), one-fourth were men (26.4%). Regarding employment status, 33.6% of the participants were employed full-time, while 30.5% were retired. The women’s unemployment rate was 21.6%, higher than the men’s unemployment rate of 11.9%. Nearly half of the participants (48.9%) were living independently with someone, with the majority being women. Only 16.4% were living with family or caregivers. A total of 127 participants (32.3%) were living alone, primarily women. Of the 82.2% of participants reporting no access to home healthcare, the percentage of men and women was nearly equal. Additionally, half of the women participants (50.8%) reported experiencing financial instability.

Concerning the disability variable, while Prolific facilitates direct data extraction from user profiles, we opted to utilize the demographic survey for data collection, acknowledging the potential for changes in disability status since profile completion. However, this method depends on self-reported participant data and warrants a cautious interpretation. To increase accuracy, we instructed the participants in the questionnaire to fill out their primary disabilities based on their diagnosis. The majority of participants (70.0%) reported having chronic physical illnesses (e.g., cardiovascular disease, cancer, diabetes, osteoarthritis, hypertension, osteoporosis), followed by mental illnesses (e.g., anxiety, depression, post-traumatic stress) at 40.0%. Participants had the option to select multiple conditions for this question. Other examples of reported disabilities include autism spectrum disorders, seizure disorders, kidney disease, low back pain, stroke, spinal damage, cerebral palsy, Parkinson’s Disease, multiple sclerosis, spine disorders, and traumatic brain injuries. Additional demographic characteristics are presented in Table 1.

### 2.2. Procedure

A cross-sectional quantitative design was utilized to address the research questions, with data collected through an online Qualtrics survey platform in April 2023. Following ethical approval from the Institutional Review Board of Michigan State University (IRB #00008515, 3 February 2023), invitations to participate were distributed via Prolific, a platform selected for its established reliability and transparency in social science research. The electronic informed consent form was embedded on the Qualtrics survey landing page, requiring participants to read and confirm their consent by selecting “Yes” to proceed with the survey. Participants were included based on two criteria: having a disability and being between 50 and 100 years old. These criteria were implemented using Prolific’s native filtering tools, which identified 4098 eligible individuals. From this pool, 400 participants were randomly selected without any stratified sampling through the Prolific platform. It should be noted that Prolific uses a first-come, first-served mechanism; the first 400 participants filled out the survey after receiving the study invitation. Given the potential for selection bias, a circumspect interpretation of the results is advised. Each participant received a reward of USD 5.08, calculated by Prolific, to ensure fair compensation for their time.

### 2.3. Measures

#### 2.3.1. Attitudes Toward Aging

The Attitudes Towards Aging Questionnaire Short Form (AAQ-SF) was used to assess participants’ attitudes toward aging. The foundational data for the Attitudes Towards Aging Questionnaire (AAQ) stems from comprehensive research supported by the European Commission’s Fifth Framework and the WHO Quality of Life Group [40]. The AAQ-SF consists of 12 items, with 4 items dedicated to each of the subscales: physical change, psychosocial loss, and psychological growth. Participants provide self-reported answers on a scale from 1 (strongly disagree) to 5 (strongly agree) for the first three items, while the remaining items (4 to 12) utilize a scoring range from 1 (not at all true) to 5 (extremely true) [41]. The short version was selected due to its strong psychometric properties, which are comparable to the original version, and its benefit in minimizing survey fatigue among participants. In the study conducted by Low et al. [42], the AAQ-SF demonstrated strong internal consistency, with reliability scores of 0.72 for the psychosocial loss and physical change subscales and 0.62 for the psychological growth subscale. In the current study, Cronbach’s alpha was calculated to be 0.81, 0.79, and 0.75 for physical change, psychosocial loss, and psychological growth, respectively.

#### 2.3.2. Purpose in Life

The Purpose in Life Test Short Form (PIL-SF) [43] was utilized to assess participants’ sense of purpose in life. The PIL-SF employs a 7-point Likert-type response format, where a score of 7 indicates a stronger sense of purpose. The overall scoring for the PIL-SF ranges from a minimum of 4 to a maximum of 28, with higher scores suggesting a greater sense of purpose in life [43]. Compared to the full version of the PIL, the PIL-SF has demonstrated reliability, with a reliability coefficient of 0.86 for the items included in a larger questionnaire and 0.84 when administered independently. In the current study, Cronbach’s alpha was calculated to be 0.91.

#### 2.3.3. Mindfulness

The Mindfulness Attention Awareness Scale (MAAS), developed by Brown and Ryan [44], was used to assess mindfulness. This scale comprises 15 items, each rated using a 6-point Likert scale. The MAAS measures an individual’s awareness of the present moment, with response options ranging from 1 (almost always) to 6 (almost never). A higher score indicates a greater level of mindfulness. The total score on the MAAS ranges from 15 to 90 [44]. The authors reported strong internal consistency, with a reliability coefficient exceeding 0.80 and a test-retest reliability score of 0.81. In the current study, Cronbach’s alpha was calculated to be 0.92.

#### 2.3.4. Acceptance of Chronic Health

The Acceptance of Chronic Health Conditions Scale (ACHC), developed by Stuifbergen and colleagues [45], was utilized to measure participants’ level of acceptance regarding their chronic health conditions or disabilities. This scale comprises 10 items, with each rated on a scale from 1 to 5. Four items required reverse coding (namely, items 1, 4, 6, and 8). The total possible score ranges from 10 to 50, with higher scores indicating a greater acceptance of chronic health conditions. In their study, Stuifbergen et al. [45] reported good internal consistency, with Cronbach’s alpha coefficients of 0.82 for individuals with poliomyelitis and 0.81 for those with multiple sclerosis. In the current study, Cronbach’s alpha was found to be 0.82.

#### 2.3.5. Loneliness

The Three-Item Loneliness Scale (T-ILS), developed by Hughes and colleagues [46], was utilized to evaluate the perceived loneliness of participants in this study. This scale comprises three distinct items, each with response options ranging from 1 to 3. Consequently, the overall score can range from a minimum of 3 to a maximum of 9, with higher scores indicating a greater degree of perceived loneliness. In their original research, Hughes et al. [46] reported that the T-ILS demonstrated good internal consistency and a strong correlation (*r* = 0.82) with the original loneliness scale. The T-ILS exhibited excellent reliability in the present study with a Cronbach’s alpha of 0.93.

### 2.4. Statistical Analysis

All variables were examined for outliers and abnormalities. The data met the assumptions of normality (skewness ranging from 0.13 to 0.88; Kurtosis ranging from 0.16 to 1.24). A Pearson correlation analysis was conducted to address concerns of multicollinearity. The results indicated that no correlation between any two variables exceeded 0.58, and the Variance Inflation Factor (VIF) for all variables ranged from 1.07 to 1.48, suggesting no multicollinearity concerns [47]. Following this, basic descriptive statistics, including percentages, ranges, means, and standard deviations, were calculated to summarize the study variables. Pearson’s correlation coefficients were computed to assess the associations among demographic variables (i.e., age, employment, marital status, and financial stability), the three subscales of attitudes toward aging, and psychological variables, including purpose in life, mindfulness, acceptance of chronic health conditions, and loneliness. An independent *t*-test was conducted to analyze the mean differences in key variables between women and men. Additionally, reflecting on previous literature reporting that gender could play a role in forming attitudes toward aging [12,19,20], we decided to run separate multiple regression analyses for men and women. This approach enables a deeper understanding of how demographic and psychological factors subtly influence attitudes toward aging, uncovering insights that may otherwise go unnoticed.

## 3. Results

### 3.1. Descriptive and Correlation Analysis

The means and standard deviations of each study variable in men and women are presented in Table 2. The analysis showed a significant difference in the physical change subscale of attitude toward aging between men and women (*p* = 0.03), with men reporting higher scores than women. Women reported significantly higher scores in psychological growth compared to their men counterparts (*p* < 0.001). There was a marginally non-significant difference in mindfulness scores between the two groups (*p* = 0.06). However, no significant differences were observed between men and women regarding the psychosocial loss scale of attitudes toward aging, purpose in life, acceptance, and loneliness.

In the correlation analysis (Table 3), purpose in life was positively correlated with physical change (*r* = 0.46, *p* < 0.001) and psychological growth (*r* = 0.58, *p* < 0.001) and negatively correlated with psychosocial loss (*r* = −0.50, *p* < 0.001). Mindfulness was significantly associated only with psychosocial loss (*r* = −0.17, *p* < 0.001). Acceptance demonstrated a significant positive correlation with positive change (*r* = 0.33, *p* < 0.001) and psychological growth (*r* = 0.26, *p* < 0.001), alongside a significant negative correlation with psychosocial loss (*r* = −0.38, *p* < 0.001). Additionally, loneliness had a negative correlation with both physical change (*r* = −0.26, *p* < 0.001) and psychological growth (*r* = −0.31, *p* < 0.001), while a strong positive correlation with psychosocial loss (*r* = 0.58, *p* < 0.001).

### 3.2. Regression Analysis

In the regression model of men (Table 4), the demographic and psychological variables accounted for 24.0% of the variance in physical change (adjusted R^2^ = 0.24; *F* (10, 132) = 5.38; *p* < 0.001), 49.0% of the variance in psychosocial loss (adjusted R^2^ = 0.49; *F* (10, 132) = 14.39; *p* < 0.001), and 38.0% of the variance in psychological growth (adjusted R^2^ = 0.38; *F* (10, 132) = 9.62; *p* < 0.001). Among these variables, purpose in life was the significant predictor for all three subscales of attitudes toward aging. It positively contributed to physical change (*β* = 0.45, *p* < 0.001) and psychological growth (*β* = 0.69, *p* < 0.001), while negatively contributed to psychosocial loss (*β* = −0.40, *p* < 0.001). Additionally, acceptance significantly contributed to lower psychosocial loss (*β* = −0.18, *p* = 0.01), whereas loneliness contributed to higher psychosocial loss (*β* = 0.43, *p* < 0.001).

In women participants (Table 5), the demographic and psychological variables explained 30.0% of the variance in physical change (adjusted R^2^ = 0.30; *F* (10, 239) = 11.87; *p* < 0.001), 35.0% of the variance in psychosocial loss (adjusted R^2^ = 0.35; *F* (10, 239) = 14.57; *p* < 0.001), and 32.0% of the variance in psychological grow (adjusted R^2^ = 0.32; *F* (10, 239) = 12.92; *p* < 0.001). Among demographic variables, age was found to significantly predict psychosocial loss (*β* = 0.15, *p* = 0.02). Regarding employment, compared to full-time employment, unemployment was associated with decreased psychosocial loss (*β* = −0.19, *p* = 0.003). Additionally, being financially stable significantly contributed to higher physical change (*β* = 0.26, *p* < 0.01). Among psychological variables, the purpose in life was a significant predictor for all three subscales of attitude toward aging. It positively contributed to physical change (*β* = 0.28, *p* < 0.001) and psychological growth (*β* = 0.53, *p* < 0.001), while negatively contributed to psychosocial loss (*β* = −0.35, *p* < 0.001). Similarly, acceptance also predicted three components of attitude toward aging. Acceptance positively predicted physical change (*β* = 0.21, *p* < 0.001) and psychological growth (*β* = 0.15, *p* = 0.01), while negatively predicted psychosocial loss (*β* = −0.24, *p* < 0.001). Furthermore, loneliness contributed to higher psychosocial loss (*β* = 0.28, *p* < 0.001).

## 4. Discussion

The current study aims to investigate pertinent demographic and psychological predictors of aging attitudes among aging adults with disabilities, as well as potential gender differences in attitudes among these individuals. Though attitudes toward aging are often used as predictors in studying outcomes, our study aims to understand precursors that lead to positive or negative attitudes. The objective is to take a step further back to preventive measures to promote changes that foster positive attitudes, ultimately influencing both aging and health outcomes.

The results from our study show that attitudes toward aging are conceptualized as a multi-faceted dimension, which is consistent with the current literature [48,49]. Our study utilized Laidlaw and researchers’ measures to capture the multidimensional aspects of aging [40,41]. The life-span development theory [50] defines the aging process as involving both the growth and decline aspects across multiple domains, which include physical function, social connectedness, as well as psychological transitions. Our correlational data show that these three dimensions of physical change, psychosocial loss, and psychological growth are significantly correlated with purpose in life, acceptance of chronic illness, and loneliness, which is supported by the current literature. For instance, AshaRani et al.’s [29] study demonstrates that a strong sense of purpose in life helps to mitigate the impacts of health-related risk factors, promoting positive attitudes and successful aging among older patients. Older adults with late-onset disabilities who accepted their disabilities as part of the aging process reported a sense of successful aging [14]. Uchmanowicz et al. [26] found that aging adults with chronic obstructive pulmonary disease reported higher scores in loneliness, among others, and also reported negative attitudes toward aging.

It is worth pointing out that the construct of psychosocial loss is significantly correlated with all four psychological predictors (i.e., purpose in life, mindfulness, acceptance of chronic illness, and loneliness), while only three of the predictor variables (not mindfulness) predict physical change and psychological growth. Therefore, the construct of psychosocial loss of aging attitudes seems to address a much more complex concept that captures not only two aspects (i.e., psychological and social) of aging but also the negative valence of loss. For one, this is consistent with what we discussed earlier, that aging is a multidimensional construct that is not only influenced by self-perception but also is a highly social concept [48,49]. Furthermore, we speculate that more factors contribute to psychosocial loss than psychological growth due to the fact that the consequence or effect of loss may be impacted differently than psychological gain, and the mechanism may be different. It is plausible that the work associated with recovering from psychosocial loss as a result of aging can be more complex than the therapeutic gain from psychological growth.

Purpose in life, acceptance of a disability or chronic health condition, loneliness, and mindfulness contributed differently toward the various aspects of aging. This is possibly due to how these psychological phenomena serve different mechanisms. Purpose in life serves to affect growth by having individuals explore the meaning of events after some changes, i.e., adverse events. This future-focused mechanism, therefore, tends to affect psychological growth the most. For instance, aging adults who have a stronger sense of purpose and meaning in life also reported more positive views about aging [32], social, economic, and health [30], and subjective well-being [31].

Acceptance of a disability, a concept first introduced by Wright [25], was used to understand the adaptation of disability for those with physical disabilities. In this concept, Wright postulated four values that describe a better adaptation through enlarging the scope of values, reducing the importance of physical appearance and focusing on other assets, viewing disability as one aspect and embracing it, and transforming the comparative values to asset values [25]. Therefore, the mechanism of acceptance of disability is through cognitive reappraisal and coping, and is likely to have the most impact on the physical changes or adaptations associated with aging attitudes. Ample studies have shown that when an aging individual with a disability comes to terms with and embraces their disability, they also have better health and mental health outcomes [27,28]. For instance, Bassett et al. [27] indicated that older women with peripheral vascular diseases do not take their illness as a loss or decline but rather accept their disability by adapting to the physical, mental, and social changes that come with growing older with a disability. Uittenhove et al. [51] further indicate that their aging participants who used emotional and acceptance coping have higher psychological strength and well-being. Thus, the ability to cognitively appraise the challenges associated with health decline and work toward adaptive coping benefits aging individuals by reducing their psychological resistance.

Loneliness operates with the element of social disconnection; therefore, it is likely to have the most influence on psychosocial loss. For instance, Lee et al. [39] reported that loneliness in aging adults is associated with perceived social loss, identity erosion, and negative aging attitudes. Kyaw et al. [52] showed that loneliness in those with cognitive impairments is associated with a faster decline in cognitive function and emotional function, contributing to their perspective on aging. In a scoping review, Fakoya et al. [53] concluded that interventions targeting loneliness in aging adults improve their perception of aging and have a better view of self (identity) as well as mood. 

Another important point to highlight is the intersectionality of aging and chronic illnesses or disabilities, as our study included older adults with these conditions. The intersectionality of aging and disability is noteworthy, as there are some common contributing factors that can lead to negative health outcomes. Relatedly, aging is likely to exacerbate one’s health concerns, while having a disability or a chronic health condition is expected to affect one’s attitudes, perceptions, or behavior in the aging process. For instance, when an aging individual is faced with a disability or a chronic illness, this disability factor is likely to amplify the aging process [4,14]. In their study, Fong et al. [15] showed that their aging participants with a chronic illness or disability perceive that the presence of a chronic illness or disability exacerbates the aging process and makes the management of the chronic health condition of multiple sclerosis more challenging. In Offermann et al.’s [11] study, their aging participants with health concerns perceive that aging affects them more negatively toward their health than those without health concerns. Furthermore, in Han’s study [16], aging adults with illnesses reported that they not only attribute their illnesses to aging but also experience more adverse health effects. Individuals with fewer chronic health issues, limited functional impairments, and lower symptoms tend to have a more positive outlook on aging [17]. Conversely, aging individuals who report positive attitudes toward aging are associated with faster recovery from severe illnesses or disabilities, fewer chronic health issues, fewer functional issues, and higher quality of life [6,7,20]. Overall, this evidence supports the trend that attitudes in the aging process can affect one’s overall health and behaviors, attesting to the importance of positive attitudes toward aging and their collateral effects on other psychosocial outcomes.

In terms of gender differences, men scored significantly higher in physical change, while women scored significantly higher in psychological growth. This difference in attitude can be attributed to sociocultural factors, including roles and expectations that society assigns to different genders [21]. Social role theory postulates that attitudes and behaviors of men and women are shaped by the societal roles they fulfill, which are often accompanied by distinct responsibilities within family and society, reinforcing gender-specific attitudes that align with prevailing social norms [22].

The above result is inconsistent with the existing literature. Women are shown to be more vulnerable to negative self-perceptions of aging due to stronger internalization of societal age norms and physical appearance standards [54], as well as social invisibility [55]. Women reported negative aging in relation to appearance and relational roles. Social media and societal messages about aging continue to reinforce gender stereotypes, which disproportionately affect women’s aging identity and expectations [56]. It is plausible that women in this study may not be as affected by their traditional gender roles. The educational and employment percentages are comparable between men and women. Evidence shows that working beyond the retirement age can have some benefits, including positive psychological well-being. A systematic review by Baxter et al. [57] indicated such positive benefits of working beyond the retirement age. However, benefits are most likely for men who work part-time in jobs that are not low in quality or reward. The conclusion attested that meaningful employment contributed positively to the positive outcomes for the aging workers. It is possible that in modern times, meaningful work can equally benefit aging women, as it also helps to promote their role identity. Furthermore, evidence shows that women often display higher resilience [58]. Therefore, these intrapersonal attributes, such as emotional resilience, can buffer against adversities and result in our women participants having more positive outcomes.

Regression analyses also showed psychological attributes (purpose in life, mindfulness, acceptance, and loneliness) collectively have a relatively even impact on aging attitudes for women with disabilities. Whereas for men, these factors had the most impact on psychosocial loss, followed by psychological growth, and the least on physical change. This points to a differential impact on the attitudes of men and women toward aging. Psychosocial factors, such as purpose in life, acceptance of chronic illness, and loneliness, can act as coping mechanisms in buffering challenges in life, such as coping with a disability and/or aging. 

Studies showed that women more frequently use emotion-focused and support-seeking coping, which may protect against the negative impact of aging stressors, while men may struggle more with role loss. For instance, Caetano et al. [59] conducted a longitudinal study of coping skills in aging individuals and found that older women were more likely than men to engage in emotion-focused coping, such as seeking emotional support, reappraisal, and acceptance, in response to aging-related losses and changes. Milner et al. [60] found that women with strong intergenerational ties and community engagement had significantly more positive views on aging. Another study showed that women were found to be more flexible in their coping approaches and more likely to apply emotion regulation and positive reframing strategies when facing aging challenges [61]. Okamoto and Tanaka [62] reported that while social support benefits both aging men and women, the benefits are more substantial for men. 

Furthermore, social networks and emotional closeness are more prominent predictors of positive aging attitudes in women. Men may benefit more from spousal support but are more vulnerable to social isolation post-retirement. Thus, it ties back to the earlier discussion that men’s perception of aging tends to focus on and be affected by the physical aspect. Men, therefore, tend to enjoy greater societal authority in later life and are more negatively impacted by physical decline and role loss. Women are more affected by appearance-related and relational factors. However, they tend to cope more adaptively through social resources related to emotional resilience. These differences underscore the importance of gender-sensitive policies and interventions in supporting healthy aging.

Findings from both the men’s and women’s data further highlight key points we would like to emphasize. First, purpose in life remains a significant factor in predicting physical change, psychosocial loss, and psychological growth for both genders. Acceptance of chronic illness and loneliness are significant predictors of psychosocial loss in aging men. Acceptance of chronic illness is a significant predictor of physical change, psychosocial loss, and psychological growth for aging women. Furthermore, loneliness is a significant predictor of psychosocial loss for aging women. Collectively, these constructs represent what Pruchno’s [63] comprehensive review on successful aging construct describes as a group of contributing factors in successful aging that is psychosocial in nature. Even though the study of successful aging in relation to having a chronic illness is not the focus of the current study, ample evidence supports that these psychosocial factors are imperative for successful aging alone, as well as for fostering positive attitudes and health outcomes. For instance, AshaRani et al. [29] found that a strong sense of purpose in life mitigated the impacts of health-related risk factors, thus promoting positive attitudes and facilitating the experience of successful aging. Guimond et al. [64] revealed that older adults who reported having a higher sense of purpose in life correlated with a lower risk of inflammation. Similarly, accepting aging and chronic illness are both shown to have beneficial effects. Uchamnowicz et al. [26] showed that among older adults with chronic obstructive pulmonary disease, poor acceptance of the disease is associated with physical fatigue, social challenges, and loneliness, leading to negative attitudes. Older adults with late-onset disabilities with a more positive level of acceptance of their disabilities also have more successful aging outcomes [14]. As for loneliness, this evidence from the disability and aging studies further supports the trend of our study that psychosocial factors, such as acceptance of chronic illness, having a purpose in life, and not being lonely in the sense of feeling engaged, play a crucial role. As Pruchno [63] concluded, in both the qualitative and quantitative aging literature, what has been consistently pointed to as a common denominator of successful aging, regardless of whether one has a disease or not, is active engagement in life.

One factor that did not show a significant result is mindfulness. Though ample literature shows that the practice of mindfulness improves positive perception about aging, physical health, and emotional well-being [34], increases cognitive performance [35] and cognitive flexibility [65]), and reduces age-related rumination [34], there are factors that mindfulness may work against the improvement of aging attitudes. The practice of mindfulness requires sustained attention and cognitive ability while aging individuals tend to have increased cognitive challenges, such as decreased processing speed, working memory, and attention, as part of the normal aging process [66]. Thus, the therapeutic progress is generally slower for individuals with cognitive or functional impairments. Many older adults may have difficulty noticing and identifying internal experiences; therefore, mindfulness practice should be guided by qualified professionals [67]. Importantly, our study assessed dispositional mindfulness rather than mindfulness practices. Future research could explore how engaging in mindfulness practices might change attitudes toward aging and, in turn, impact health outcomes in this population.

### 4.1. Limitations of the Study and Future Directions

While this study investigated potential predictors of attitudes toward aging among aging adults with disabilities, which contribute to the general literature on aging, chronic illness, and gender aspects, it is not without limitations. Although this study has a relatively large sample size, the types of chronic illnesses and disabilities are not homogeneous; thus, it is plausible that attitudes and other studied psychosocial factors may manifest differently. While we attempt to focus on chronic illness/disability groups, not having a control group to compare is another limitation. Additionally, the overlap and intersectionality of aging and disability could be better studied and analyzed using either a different research methodology or statistical methodology. Given that the aging process is complex and aging attitudes can change over time, influenced by a multitude of factors, a longitudinal study would more accurately capture accurate outcomes than a cross-sectional study. Although our participants reported different types of disabilities, the number of participants in each disability category is not adequate to warrant further analysis of the differences in aging attitudes and how psychological factors influence their attitudes differently. Thus, future studies could focus on specific types of disability or compare different disabilities. Furthermore, data collection through Prolific may limit the generalizability of findings, as the participant pool tends to comprise younger individuals with higher education, greater digital literacy, and better overall health, potentially resulting in the underrepresentation of specific populations. Finally, all survey questions, including those pertaining to demographics like disability status, rely on self-reported data, which may exhibit inherent limitations in objectivity and accuracy. Thus, all these limitations guide future researchers in expanding this work.

### 4.2. Implications of Findings in Practice and Research

Given that our studies showed differences in aging attitudes and the psychosocial factors affecting these attitudes, we advocate for gender-specific applications in clinical practice. Aging women can benefit from emotion-based, social, and appearance-focused support, while men can benefit from control-based, purpose-driven, and identity-reframing approaches. Specifically, strategies that foster positive aging identity, particularly through peer support and workshops that reduce internalized ageism by disputing media stereotypes, promoting positive body image and self-compassion [68], and reframing aging as signs of growth, wisdom, and intergenerational value [69] are recommended. Relatedly, studies show that women who remain socially and intergenerationally engaged have more positive views on aging. Thus, the involvement of older women in the role of mentoring younger generations and public education roles can promote change not only in individuals but also in societal attitudes and traditional roles [70]. In addition, women can benefit from the use of emotion-focused coping strategies, such as emotional reappraisal and expression, to enhance meaning-making and emotional regulation [59].

For aging men, the reframing of masculinity and loss of physical function can be achieved by redefining masculinity in terms of wisdom, mentorship, and emotional availability [71]. Men’s role identity can be refocused from work roles to other meaningful roles [72]. Furthermore, helping aging men achieve mastery and control over their health can be crucial; thus, providing practical tips and workshops on health-focused behaviors and health-tracking activities with individualized goals can be helpful in promoting positive attitudes and health outcomes [73]. In addition, supporting aging men in normalizing emotional disclosure and expression within men-oriented spaces may be valuable.

In terms of research implications, what remains imperative is to continue conducting research to understand the complexity of aging, how aging attitudes affect health and well-being outcomes, and how the intersectionality of aging and chronic illnesses and disabilities interplay. The use of more advanced statistics (e.g., latent profiling) and/or research methodology (e.g., longitudinal design) to better capture the effects of aging across time is of importance. Research on addressing the effects of aging on different populations is another area that needs attention. Given that our participants include individuals with disabilities, comparing these findings with those of individuals without disabilities would provide valuable insights into how disability status intersects with gender and aging to influence attitudes. The wisdom and success of successful aging offer researchers much to learn from those who demonstrate positive aging attitudes and age successfully.

## 5. Conclusions

The current study aimed to investigate gender differences in attitudes toward aging among individuals with disabilities while also examining the influence of demographic and psychological factors on these attitudes. The results showed some significant differences in physical change and psychological growth between men and women, with men reporting more positive attitudes toward physical change and women toward psychological growth. Additionally, factors influencing attitudes also differed by gender, with purpose in life being the strongest factor predicting all domains of attitudes among both men and women. Interestingly, demographic factors such as age, employment, and financial stability were significant only among women. These findings underscore the crucial role of gender in shaping aging attitudes and their psychological factors, emphasizing the need for gender-specific interventions that address distinct challenges and psychological resources within each group. Further research, particularly using longitudinal and comparative designs, is essential to deepen our understanding of the complexity of aging, including how the intersectionality of gender, disability status and types, and aging attitudes influence health and well-being in older adults over time.

## Figures and Tables

**Table 1:** Demographic characteristics of participants (*N* = 393).

Variables	Total Sample (%)	Men (%)	Women (%)
Age			
Below 65	286 (72.8)	105 (73.2)	181 (72.4)
65 or above	107 (27.2)	38 (26.6)	69 (27.6)
Race/ethnicity			
White	340 (86.5)	124 (86.7)	216 (86.4)
Black	25 (6.4)	7 (4.9)	18 (7.2)
American Indian or Alaskan Native	4 (1.0)	1 (0.7)	3 (1.2)
Asian	7 (1.8)	6 (4.2)	1 (0.4)
Mexican American	3 (0.8)	1 (0.7)	2 (0.8)
Puerto-Rican	1 (0.3)	1 (0.7)	-
From multiple races	6 (1.5)	1 (0.7)	5 (2.0)
Others	7 (1.8)	2 (1.4)	5 (2.0)
Marital status			
Never married	77 (19.6)	32 (22.4)	45 (18.0)
Married or with a partner	183 (46.6)	79 (55.2)	104 (41.6)
Divorced or separated	99 (25.2)	25 (17.5)	74 (29.6)
Widowed	34 (8.7)	7 (4.9)	27 (10.8)
Education			
Less than a high school degree	2 (0.5)	-	2 (0.8)
High school degree or equivalent	48 (12.2)	18 (12.6)	30 (12.0)
Some college but no degree	99 (25.2)	33 (23.1)	66 (26.4)
Associate degree	57 (14.5)	19 (13.3)	38 (15.2)
Bachelor’s degree	120 (30.5)	44 (30.8)	76 (30.4)
Graduate degree	67 (17.0)	29 (20.3)	38 (15.2)
Employment status			
Full-time employed	132 (33.6)	59 (41.3)	73 (29.2)
Part-time employed	70 (17.8)	19 (13.3)	51 (20.4)
Not employed, currently looking for work	24 (6.1)	5 (3.5)	19 (7.6)
Not employed, not looking for work	47 (12.0)	12 (8.4)	35 (14.0)
Retired	120 (30.5)	48 (33.6)	72 (28.8)
Current living situation			
Living independently alone	127 (32.3)	43 (30.1)	84 (33.6)
Living independently with someone	192 (48.9)	74 (51.7)	118 (47.2)
Living with family or other caregivers	64 (16.3)	22 (15.4)	42 (16.8)
Assisted living facility	2 (0.5)	1 (0.7)	1 (0.4)
Others	8 (2.0)	3 (2.1)	5 (2.0)
Accessibility of home healthcare			
No	323 (82.2)	116 (81.1)	207 (82.8)
Yes	70 (17.8)	27 (18.9)	43 (17.2)
Financial stability			
Strongly agree	25 (6.4)	14 (9.8)	11 (4.4)
Agree	132 (33.6)	60 (42.0)	72 (28.8)
Neutral	66 (16.8)	26 (18.2)	40 (16.0)
Disagree	104 (26.5)	26 (18.2)	78 (31.2)
Strongly disagree	66 (16.8)	17 (11.9)	49 (19.6)
Primary disability types (multiple responses)			
Chronic physical illnesses	275 (70)	94 (65.7)	181 (72.4)
Mental illnesses	158 (40)	52 (36.4)	106 (42.4)
Dementia	2 (0.5)	1 (0.7)	1 (0.4)
Substance use disorders	12 (3.1)	4 (2.8)	8 (3.2)
Others	69 (17.6)	20 (14.0)	49 (19.6)
Prefer not to answer	17 (4.3)	11 (7.7)	6 (2.4)

**Table 2:** Descriptive statistics for continuous variables and independent *t*-tests.

Variables	Total Sample Mean (SD)	Men Mean (SD)	Women Mean (SD)	*T*-Test	*p*-Value
Age	60.63 (3.6)	61.31 (7.72)	59.81 (7.69)	1.86	0.06
Physical change	10.54 (4.10)	11.15 (4.13)	10.20 (4.05)	2.22	0.03
Psychosocial loss	11.79 (4.07)	12.05 (4.08)	11.56 (4.04)	1.16	0.25
Psychological growth	15.45 (3.17)	14.78 (3.20)	15.92 (3.00)	−3.55	<0.001
Purpose in life	18.82 (5.50)	18.34 (5.83)	19.22 (5.27)	−1.53	0.13
Mindfulness	3.76 (0.76)	3.87 (0.63)	3.72 (0.79)	1.92	0.06
Acceptance	26.75 (7.45)	26.32 (6.78)	26.94 (7.86)	−0.79	0.43
Loneliness	6.93 (3.56)	7.05 (3.6)	6.78 (3.41)	0.76	0.45

**Table 3:** Correlation coefficients among psychological variables and attitudes toward aging.

Variables	1	2	3	4	5	6
1. Physical change	1					
2. Psychosocial loss	−0.39 ***	1				
3. Psychological growth	0.32 ***	−0.48 ***	1			
4. Purpose in life	0.46 ***	−0.50 ***	0.58 ***	1		
5. Mindfulness	0.08	−0.17 ***	0.03	0.18 ***	1	
6. Acceptance	0.33 ***	−0.38 ***	0.26 ***	0.32 ***	0.13 **	1
7. Loneliness	−0.26 ***	0.58 ***	−0.31 ***	−0.52 **	−0.24 ***	−0.31 ***

** *p* < 0.01; *** *p* < 0.001.

**Table 4:** Regression model for predicting attitudes toward aging in men.

Predictors	Physical Change			Psychosocial Loss			Psychological Growth		
	B	*β*	*p* Value	B	*β*	*p* Value	B	*β*	*p* Value
Age	−0.02	−0.04	0.63	0.03	0.06	0.39	−0.02	−0.05	0.56
Employment									
Full time (reference)									
Part-time	−1.12	−0.09	0.26	0.40	0.03	0.62	−0.37	−0.04	0.59
Unemployed	−1.20	−0.09	0.30	−0.83	−0.07	0.38	1.01	0.10	0.21
Retired	−0.90	−0.10	0.27	−0.32	−0.04	0.63	0.46	0.07	0.42
Marital status	−0.30	−0.04	0.67	1.06	0.13	0.06	0.30	0.05	0.54
Financial stability	0.33	0.10	0.31	0.52	0.15	0.05	−0.38	−0.14	0.10
Purpose in life	0.32	0.45	<0.001	−0.28	−0.40	<0.001	0.38	0.69	<0.001
Mindfulness	0.37	0.06	0.51	−0.35	−0.05	0.44	−0.73	−0.14	0.06
Acceptance	0.09	0.14	0.10	−0.11	−0.18	0.01	0.02	0.04	0.57
Loneliness	0.16	0.14	0.15	0.48	0.43	<0.001	−0.06	−0.07	0.44
	Adjusted R^2^ = 0.24; F (10, 132) = 5.38; *p* < 0.001			Adjusted R^2^ = 0.49; F (10, 132) = 14.39; *p* < 0.001			Adjusted R^2^ = 0.38; F (10, 132) = 9.62; *p* < 0.001		

**Table 5:** Regression model for predicting attitudes toward aging in women.

Predictors	Physical Change			Psychosocial Loss			Psychological Grow		
	B	*β*	*p* Value	B	*β*	*p* Value	B	*β*	*p* Value
Age	0.05	0.09	0.18	0.08	0.15	0.02	−0.004	−0.01	0.87
Employment									
Full time (reference)									
Part-time	0.13	0.01	0.83	−0.96	−0.10	0.12	−0.55	−0.07	0.24
Unemployed	0.15	0.03	0.82	−1.87	−0.19	0.003	0.73	0.10	0.12
Retired	−0.34	−0.04	0.60	−1.16	−0.13	0.06	0.39	0.06	0.41
Marital status	−0.32	−0.04	0.51	0.44	0.05	0.35	0.06	0.01	0.88
Financial stability	0.88	0.26	<0.001	0.26	0.08	0.21	0.10	0.04	0.55
Purpose in life	0.22	0.28	<0.001	−0.27	−0.35	<0.001	0.30	0.53	<0.001
Mindfulness	−0.33	−0.07	0.23	0.01	0.001	0.98	−0.20	−0.05	0.32
Acceptance	0.11	0.21	<0.001	−0.12	−0.24	<0.001	0.06	0.15	0.01
Loneliness	−0.06	−0.05	0.43	0.33	0.28	<0.001	0.004	0.005	0.94
	Adjusted R^2^ = 0.30; F (10, 239) = 11.87; *p* < 0.001			Adjusted R^2^ = 0.35; F (10, 239) = 14.57; *p* < 0.001			Adjusted R^2^ = 0.32; F (10, 239) = 12.92; *p* < 0.001		

## Data Availability

This study is still ongoing, so the data that support the findings of this study are available from the corresponding author, M.B., upon reasonable request.
